# Development of Enzyme-Linked Immunosorbent and Immunochromatography Assays for Diagnosing *Nosema ceranae* Infection in Honey Bees

**DOI:** 10.3390/insects15010059

**Published:** 2024-01-13

**Authors:** Jae Kwon Lee

**Affiliations:** Department of Biology Education, College of Education, Chungbuk National University, Cheongju 28644, Republic of Korea; chemokine@chungbuk.ac.kr

**Keywords:** *Nosema ceranae*, monoclonal antibodies, honeybee, nosemosis, sandwich ELISA

## Abstract

**Simple Summary:**

*Nosema ceranae* (*N. ceranae*), a highly aggressive pathogen that affects honeybees, can rapidly spread infection under suboptimal conditions for honeybee breeding, consequently reducing honey production. Preventing and treating *Nosema* infection remains challenging since symptoms are often manifested in bees only after several million *Nosema* spores are present in the bee midgut. In this study, specialized methods, namely a sandwich enzyme-linked immunosorbent assay (ELISA) and an immunochromatography assay (ICG), were developed for diagnosing *N*. *ceranae* infections, and their effectiveness in diagnosing *Nosema* infections was evaluated. The sandwich ELISA method effectively detected *Nosema* infection; comparatively, the ICG approach was less efficient. This comparison between the two diagnostic methods provides insights into their respective strengths and limitations, facilitating future investigations and potential improvements in the development of more effective diagnostic approaches for detecting and managing *N*. *ceranae* infections in honeybees.

**Abstract:**

*Nosema ceranae* (*N. ceranae*) infection is prevalent globally, causing a decline in bee populations and significant economic losses to apiarists. Although several methods have been proposed for diagnosing *Nosema* infections, limitations in these methods have hindered their broad applications. Therefore, this current study aimed to develop a specialized method for diagnosing *Nosema* infections. To achieve this, a sandwich enzyme-linked immunosorbent assay (ELISA) and immunochromatography assay (ICG) were developed, and their effectiveness in screening and diagnosing *Nosema* infection was assessed. In sandwich ELISA, the combination of the monoclonal antibodies (mAb) 19B2 and biotinylated-19B2 exhibited stronger binding affinity to the antigen than did other combinations of mAbs that were tested. Furthermore, the antigen detection limit achieved with the sandwich ELISA surpassed that previously reported with Western blotting. The ICG was designed using the same antibody combination as that used in sandwich ELISA; however, the assay exhibited a lower diagnostic ability for *Nosema* infection than the ELISA. The diagnostic models developed in this study offer practical applications for conducting rapid nosemosis detection tests. These innovative techniques will help to improve the timely identification and management of nosemosis.

## 1. Introduction

*Nosema apis* and *Nosema ceranae* are causative agents of nosemosis, one of the most prevalent diseases among honeybees. These unicellular parasites invade the midgut of honeybees, debilitating individual bees and entire colonies, rendering them more susceptible to various pests and diseases [1]. Over the last two decades, *N. ceranae* has rapidly invaded *Apis mellifera* populations, establishing a global presence and posing a more substantial threat to honeybee health than that caused by *N. apis* [2,3,4,5,6]. Moreover, even if the bees are uninfected, millions of *N. ceranae* spores can persist in beekeeping equipment. Given the ubiquitous presence of this pathogen, infection levels can rapidly escalate under suboptimal honeybee breeding conditions, thereby diminishing honey production and, in severe instances, causing colony collapse.

The primary challenge in preventing and treating *Nosema* infection is that by the time symptoms are manifested in bees, there are often several million *Nosema* spores present in the midgut of the bees. Diagnosing nosemosis presents a challenge since distinctive symptoms are absent. While diarrhea is often considered an initial symptom of *Nosema* infection, it is not exclusive to nosemosis [7]. Therefore, the effect of nosemosis infections may be significantly reduced using specialized diagnostic approaches.

The most frequently employed techniques for diagnosing *Nosema* infection involve directly observing spores through a microscope and confirming the presence of *Nosema* genes using a polymerase chain reaction (PCR). The microscope observation method is preferred owing to its simple sample preparation process [8,9]. However, the presence of contaminants, such as tissue fragments, dust, intestinal yeast, and other elements, during spore isolation hinders the achievement of an accurate diagnosis. PCR is a diagnostic technique capable of confirming nosemosis by detecting *Nosema* spp. genes [3,10]. However, it involves a lengthy procedure for gene isolation and requires specialized experimental procedures and specific equipment, such as a thermocycler [11]. For these reasons, there is a need for more accurate and convenient methods, such as enzyme-linked immunosorbent assay (ELISA), for diagnosing *Nosema* infections.

A previous study used three monoclonal antibodies (mAbs), namely, 9A4, 14A8, and 19B2 to target *Nosema* spores [12]. In this current study, we used these antibodies to establish a precise and convenient diagnostic method for *N. ceranae*-induced nosemosis. Our results demonstrated that sandwich ELISA could effectively detect *Nosema* infection while immunochromatography (ICG) could not. A sandwich ELISA quantifies the presence of an antigen using a dual-layer of antibodies comprising capture and detection antibodies. The primary benefit of employing a sandwich ELISA lies in its remarkable binding activity to antigens, surpassing that of direct or indirect ELISA. Furthermore, sandwich ELISA has superior specificity since it relies on a pair of antibodies to identify target antigens. The utilization of sandwich ELISA can enable beekeepers to monitor and assess the prevalence of *N. ceranae* infections in their bee colonies more efficiently. By incorporating this diagnostic tool into routine beekeeping practices, beekeepers can implement timely and targeted interventions, thereby improving the overall health and productivity of their honeybee populations.

## 2. Materials and Methods

### 2.1. Isolation of N. ceranae Spores (Preparation of N. ceranae Spore Antigens)

Honeybees infected with *N. ceranae* were acquired from an apiary in Chungbuk Province, Republic of Korea. *Nosema* spores were isolated using a previously described method [13]. The honeybee midgut was thoroughly washed with phosphate-buffered saline (PBS) and homogenized using a tissue grinder. A 70-μm mesh filter was utilized to remove large particles. The suspension that passed through the filter was subjected to centrifugation at 250× *g* and 400× *g* rcf for 10 min. The isolated spores were identified using specific primers (sense strand: 5′-CGGATAAAAGAGTCCGTTACC-3′, antisense strand: 5′-TGAGCAGGGTTCTAGGGAT-3′, Bioneer Co., Daejeon, Republic of Korea) for *N. ceranae,* as previously described [9,14,15]. *Nosema* spore lysates were generated through sonication. To achieve this, *Nosema* spores were combined with RIPA lysis buffer (50 mM Tris-HCl, 150 mM NaCl, 1% NP-40, 0.5% sodium deoxycholate, and 0.1% sodium dodecyl sulfate (SDS)). The mixture was supplemented with a protease inhibitor cocktail (Sigma, St. Louis, MO, USA), sonicated, and centrifuged at 18,000× *g* rcf for 10 min.

### 2.2. Monoclonal Antibody for N. ceranae

The procedure for generating three different *N. ceranae*-targeting mAbs, specifically 9A4 (IgG1), 14A8 (IgG2b), and 19B2 (IgG3), has been described previously [12]. To obtain substantial quantities of the antibodies (2 mg/mL, total volume 3 mL), hybridoma cells were cultured in Dulbecco’s modified Eagle’s medium (DMEM; Hyclone, Logan, UT, USA) supplemented with 10% fetal bovine serum (FBS; Hyclone Laboratories, Logan, UT, USA), 100 U/mL penicillin (Gibco BRL, Grand Island, NY, USA), and 100 μg/mL streptomycin (Gibco BRL).

The anti-*Nosema* mAbs were purified by targeting IgG from 20 mL of hybridoma culture supernatants; the final mAb was resuspended in FBS-free DMEM. The process involved using a 5-mL HiTrap desalting column, followed by a 1-mL HiTrap protein G HP column, according to the manufacturer’s instructions. The mAb yield obtained was >2 mg/mL. The purified antibody was confirmed through the presence of 50 and 25 kDa bands on a 12% acrylamide gel via sodium dodecyl sulfate-polyacrylamide gel electrophoresis (SDS-PAGE) under reducing conditions.

### 2.3. Feasibility Test of Antibody

The reactivity of the mAbs with *N. ceranae* antigens was assessed following the ELISA protocol described by Anuracpreeda et al. [16]. Briefly, wells were coated with *N. ceranae* antigen, either in the form of whole spores or spore lysate diluted in a carbonate/bicarbonate coating buffer (15 mM Na_2_CO_3_, 35 mM NaHCO_3_, and pH 9.6). Following a 2 h incubation at 37 °C, the microtiter plate was washed three times with the ELISA buffer (0.05% Tween-20 in PBS). Subsequently, each well was treated with 100 μL of blocking buffer, containing the ELISA buffer and 0.5% bovine serum albumin (Sigma), and incubated for 1 h at 37 °C. After washing, 50 μL of mAb was introduced into the wells and incubated for 2 h at 37 °C. The plate was washed once more, following which 50 μL of horseradish peroxidase (HRP)-conjugated goat anti-mouse IgG (1:10,000 dilution) was added and incubated for an additional 1 h at 37 °C. After washing the plate, 50 μL of 3,3′,5,5′-tetramethyl benzidine (TMB; KPL, Gaithersburg, MD, USA) was added to each well. The process was conducted in the dark for 20–30 min at 25 °C. The reaction was terminated by adding 50 μL of stop solution (1 N HCl), and the optical densities (OD) were measured at 450 nm using a microplate reader (Bio-Rad Laboratories, Hercules, CA, USA).

### 2.4. Biotinylation of mAbs

The mAbs were conjugated to biotin molecules using a commercially available antibody biotinylation kit (Abcam, Cambridge, MA, USA), following the manufacturer’s instructions. Briefly, 10 μg of antibody were mixed with a ‘Modifier reagent’ at a 10:1 ratio and introduced to the lyophilized Biotin Conjugation Mix, followed by suspension. After incubating for >15 min in the dark at 25 °C, one-tenth of the volume of the quencher reagent was incorporated. The biotinylated mAbs were used without purification.

### 2.5. SDS-PAGE

SDS-PAGE was performed using the Laemmli method, as previously described [17]. Proteins were separated on 12% or 15% acrylamide gels, and subsequently, the gel was stained with Coomassie Brilliant Blue (Bio-Rad Laboratories).

### 2.6. Sandwich ELISA

The capture antibody was diluted to a final concentration of 1–5 μg/mL using carbonate/bicarbonate buffer (pH 7.4), from which 100 µL was added to each well of a microplate (MaxiSorp, Waltham, MA, USA). After overnight incubation at 4 °C, the plate was washed and blocked with 3% skim milk solution. Following a 2 h incubation at 4 °C, the plate was washed, and the diluted samples were introduced using a blocking buffer. After a 2 h incubation at 25 °C, the plate was washed again, and 100 µL of the biotinylated detection antibody (1 μg/mL) was added and incubated for 2 h at 25 °C. Thereafter, the plate was washed again, and a 1:5000 diluted avidin-HRP conjugate was added. After a 30 min incubation at 25 °C, the plate was washed, and 50 μL of TMB solution was added. The reaction was performed in the dark for 20–30 min at 25 °C and terminated by the addition of 50 μL stop solution (1 N HCl). Subsequently, the OD was measured at 450 nm using a microplate reader.

### 2.7. Immunochromatography (ICG) to Diagnose N. ceranae Infection

**Figure 1:**
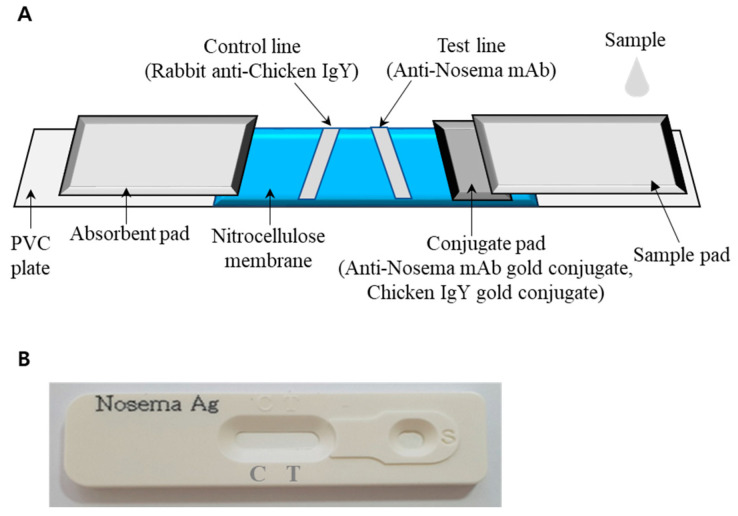
Schematic diagram of the immunochromatography (ICG) strip. (**A**) The colloidal gold test strip comprised three pads (sample, conjugate, and absorbent), a nitrocellulose membrane, and a PVC plate. The conjugate pad contained anti-*Nosema* mAb gold and chicken IgY gold conjugates, manifesting as a distinct red color. The nitrocellulose membrane displayed two lines, namely the control and test. The control and test lines comprised rabbit anti-chicken IgY mAb and mouse anti-*Nosema* mAb, respectively. (**B**) The fully packed strips enclosed within cassettes.

The ICG strip used for diagnosing *Nosema* infection was produced in collaboration with VetAll Laboratories (Goyang-si, Gyeonggi-do, Republic of Korea). Briefly, to conjugate the antibody with gold particles, the 9A4, 14A8, and 19B2 mAbs were mixed with a colloidal gold solution (Abcam) at varying concentrations (6–20 μg/mL) and with a pH range of 6–9; the pH was adjusted by adding 0.1 M K_2_CO_3_. Subsequently, the mixture was subjected to centrifugation at 15,000× *g* for 30 min to remove uncoordinated proteins. 9A4, 14A8, and 19B2 exhibited the most effective conjugation with the colloidal gold at 8 μg/mL (pH 7), 10 μg/mL (pH 6), and 10 μg/mL (pH 6), respectively. A gold-conjugated antibody (anti-*Nosema* mAb gold conjugate or chicken IgY gold conjugate) with an OD value of 3 was evenly distributed on a segment of the conjugate pad. Purified anti-*Nosema* mAb and rabbit anti-chicken IgY were then applied to nitrocellulose membranes (Immunopore membrane; Whatman, Maidstone, UK) to create the test and control lines, respectively (Figure 1A). The prepared sample pad and conjugate pad, nitrocellulose membrane, and absorbent pad were successively affixed to a PVC plate. Figure 1B illustrates the complete *Nosema* Antigen Test Kit.

### 2.8. Pairing test of Colloidal Gold Strip

To assess the activity of the colloidal gold test strip for *Nosema* infection, 9A4, 14A8, and 19B2 and three different gold conjugated mAbs (9A4, 14A8, and 19B2) were tested using the strips. The diagnostic activity of these test strips was determined based on the test line intensity values. The intensities of the control and test lines were quantified using the Image J Gel Analysis program. 

### 2.9. Detection of Nosema Spores in Experimental Specimens

To assess ICG’s feasibility, honeybees were divided into normal and weak groups, with *Nosema* infection being confirmed through microscopic observation. To prepare the test samples, we followed the spore isolation procedure previously described [13]. Briefly, *Nosema* spores were isolated by rinsing the honeybee midgut with PBS and homogenizing it with a tissue grinder. To remove large particles, a 70 µm mesh filter was utilized. Subsequently, the filtered suspension underwent centrifugation at 250× *g* and 400× *g* rcf for 10 min each. The separated spores, suspended in the diluent buffer supplied by the kit manufacturer (VetAll Laboratories), were homogenized using a homogenizer pestle (SciLab, Seoul, Republic of Korea). Subsequently, three drops of the diluted sample were dispensed into the circular window of the “*Nosema* antigen test kit.” Following the manufacturer’s guidelines, the test reaction was left to stand for 10 min, following which a visual assessment was conducted. The appearance of two red lines, which denoted the control (C) and test (T), was regarded as a positive result, indicating the presence of *Nosema* spore antigens in the sample. In contrast, if only the C line appeared, the result was considered negative; however, if line C did not appear, it was regarded as invalid.

### 2.10. Statistical Analysis

Data are expressed as means ± standard deviation. SPSS (version 17.0; SPSS Inc., Chicago, IL, USA) was used for data analysis.

## 3. Results

### 3.1. Generation of Hybridoma and Characterization of mAbs

**Figure 2:**
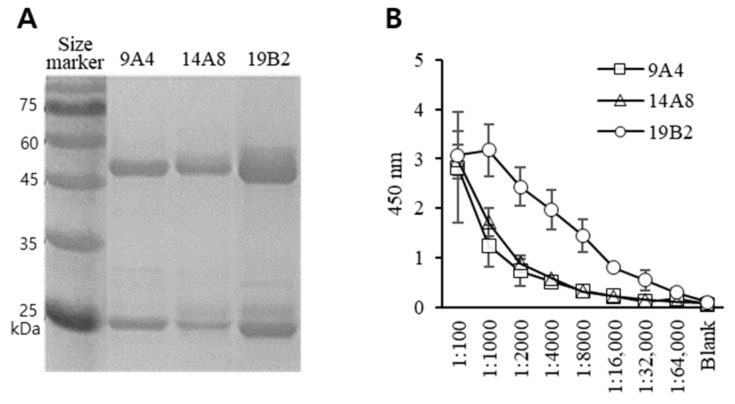
Confirmation of immunoglobulin proteins and monoclonal antibody (mAb) suitability. (**A**) Analysis of three distinct mAbs (9A4, 14A8, and 19B2) using SDS-PAGE with 12% acrylamide gel. Each acrylamide gel was subsequently stained with Coomassie brilliant blue. (**B**) Purified immunoglobulins were diluted serially and evaluated as primary antibodies, with HRP-conjugated anti-mouse IgG (1:10,000 dilution) utilized as the secondary antibody. All experiments were repeated more than thrice, and representative data are presented. Values are presented as mean ± standard deviation (n = 3).

To obtain a substantial quantity of antibodies, hybridoma cells were cultured in vitro in DMEM containing 10% FBS. mAbs were obtained from the cell culture supernatants and utilized in experimental diagnostic tests. The mAbs 9A4, 14A8, and 19B2 comprised heavy and light chain fragments of approximately 50 kDa and 25 kDa, respectively (Figure 2A), consistent with the pattern typically associated with an IgG subclass.

### 3.2. Feasibility Test of Antibody 

To demonstrate the binding activity of 9A4, 14A8, and 19B2, spore lysates at a concentration of 10 μg/mL were assessed, adhering to the ELISA protocol with mAb serial dilution. The three mAbs showed reactivity with spore lysate samples, particularly at a high concentration (1:100 dilution of 2 mg/mL purified antibody solution). When diluted to 1:2000, 19B2 exhibited a reactivity of approximately 76% compared with the highest response level (Figure 2B). Moreover, 19B2 demonstrated the highest response when *Nosema* lysate was diluted to 1:1000. Conversely, 9A4 and 14A8 exhibited reactivity of approximately 44% and 58%, respectively, when exposed to *Nosema* lysate diluted at 1:1000, compared with the highest response level.

### 3.3. Effect of mAb Biotinylation on Antibody Binding to Nosema Spore

To enhance the detection activity of mAb for *Nosema* spores, each mAb was conjugated with biotin. As shown in Table 1, both 14A8 and 19B2, irrespective of biotinylation, exhibited strong reactivity with the *Nosema* lysate in a concentration-dependent manner. Among the mAbs, both before and after biotinylation, 14A8 exhibited the most potent binding response. Furthermore, biotinylated-19B2 (average OD: 1.07) demonstrated a significant improvement in reactivity against the *Nosema* lysate than the unbiotinylated-19B2 (average OD: 0.25); a consistent pattern was observed when reacting with whole *Nosema* spores (Table 2). However, the overall reactivity of mAbs toward whole *Nosema* spores tended to be slightly lower than that toward the *Nosema* lysate, because a whole spore cannot adequately expose antigen epitopes.

### 3.4. Sandwich ELISA for Diagnosis of Nosema Infection

To establish a sandwich ELISA method, pairing tests were conducted using biotinylated and unbiotinylated mAbs. The capture antibody (i.e., unbiotinylated mAb) was coated onto microplates at a concentration of 1 μg/mL; subsequently, *Nosema* spore lysate at concentrations of 250 and 1000 ng/mL was added and incubated for 2 h, following which a biotinylated detection antibody (1 μg/mL) was added. The paring test (Table 3) findings revealed that *Nosema* lysate at a concentration of ≤ 1 μg /mL could not be detected under the tested conditions (1 μg /mL capture antibody and 1 μg/mL detection antibody). Therefore, in the subsequent experiment (Table 4), a pairing test was conducted by increasing the concentrations of the capture antibody and *Nosema* lysate. In the ELISA method, conducted with the concentration of the capture antibody set to 5 μg/mL and the *Nosema* lysate to 10,000 ng/mL, the combinations of “19B2 and biotinylated-19B2” (average OD: 0.50) and “19B2 and biotinylated-14A8” (average OD: 0.39) demonstrated meaningful binding activity against low concentration *Nosema* lysate (156.3 ng/mL). The “19B2 and biotinylated-19B2” combination demonstrated superior binding activity to the *Nosema* lysate of 1000 ng/mL (average OD: 3.05) than did the other tested combinations.

### 3.5. Application of mAbs to ICG

**Figure 3:**
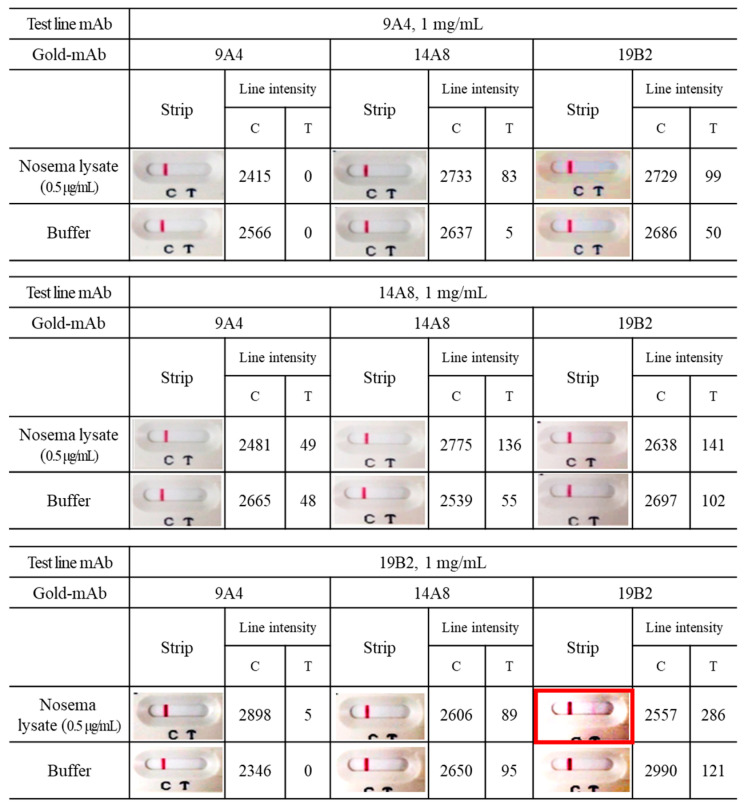
Selection of the optimal combination between gold-conjugated and unconjugated antibodies for ICG establishment. Each anti-*Nosema* mAb was reacted with three different gold-conjugated anti-*Nosema* mAbs (Gold-mAb). The diagnostic activity of the test strip was estimated based on the corresponding band intensity values (C: control line, T: test line). All experiments were repeated more than thrice, and data from one representative experiment are presented.

An ICG was developed for the rapid detection of *Nosema* infection with the same antibodies (i.e., 9A4, 14A8, and 19B2) used in the sandwich ELISA method. Following the labeling of 9A4, 14A8, and 19B2 with colloidal gold, the binding activity was assessed using the “colloidal gold test strip” to detect *Nosema* infection. To determine the most effective combination, each gold-conjugated mAb was paired with 9A4, 14A8, and 19B2. The combination of gold-conjugated 19B2 and 19B2 exhibited a stronger reaction with *Nosema* lysate (0.5 μg/mL) than did other mAb combinations, revealing the most distinct band with an intensity of 286 (Figure 3). Therefore, the 19B2 antibody pair, serving as the capture and gold-conjugated antibody, was selected for the colloidal gold test strip.

**Figure 4:**
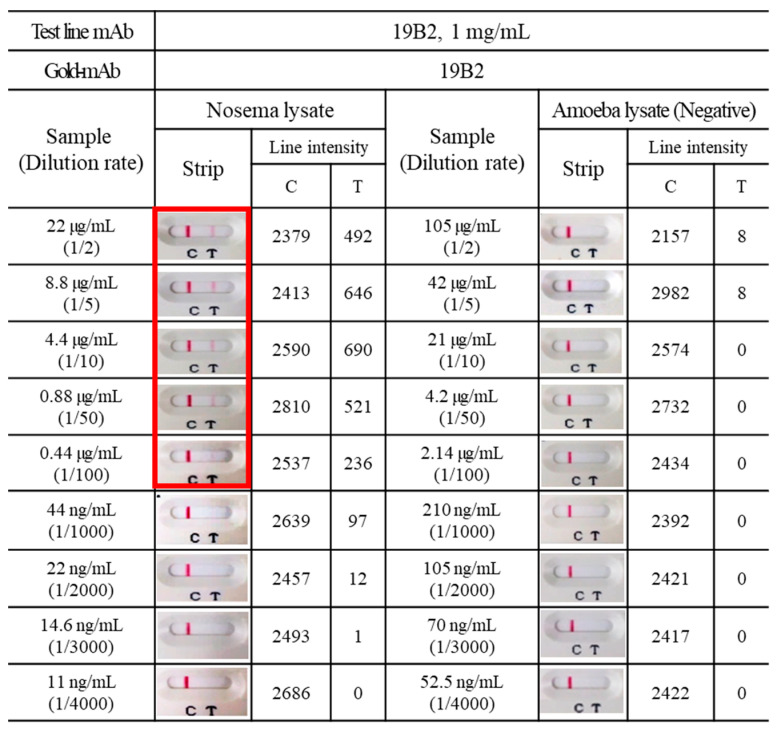
Limit of detection for the ICG strip using *N. ceranae* spore lysate. The ICG strip comprises a conjugate pad coated with gold-19B2 and a test line coated with 19B2 (1 mg/mL). The ICG results were obtained by applying various *Nosema* lysate concentrations (11–22,000 ng/mL) or Amoeba lysate (52.5–105,000 ng/mL) on the ICG strip. The intensities of the control and test lines were analyzed using ImageJ. All experiments were repeated more than thrice, and data from one representative experiment are presented.

Next, to establish the detection limit of *Nosema* infection using ICG, the presence of *Nosema* at various sample concentrations (*Nosema* lysate) was explored. Test strips that contained the gold conjugate-19B2 and 19B2 combination were utilized. Distinct bands corresponding to *Nosema* lysates were detected in samples with a total protein concentration of up to 440 ng/mL. Amoeba (*Malpighamoeba mellificae*) is a microorganism found in the honeybee midgut and shares similarities with *Nosema* spores (Figure 4). Therefore, amoeba lysates were used as analogous but distinct antigens to *Nosema* spp. The ICG designed for *Nosema* did not detect the amoeba lysate, even at its highest concentration. 

**Figure 5:**
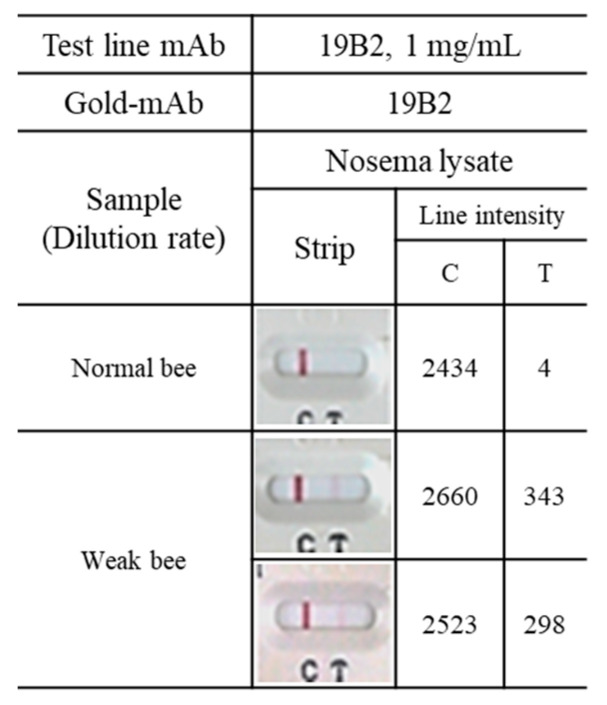
Detection of *Nosema* spore in experimental specimens.

Figure 5 shows the results of *Nosema* infection testing in bees collected from an apiary in Chungbuk Province. Weak bees exhibited discernible red lines in the *Nosema* diagnostic kit test, and laboratory microscopy revealed that each weak bee had approximately 3 × 10^6^ *Nosema* spores.

## 4. Discussion

Nosemosis remains a major threat to honeybee populations, and beekeepers frequently struggle to achieve accurate diagnoses before administering anti-*Nosema* medications [3,7]. The diagnosis of *Nosema* spores serves a dual purpose: safeguarding honeybee colonies against nosemosis and preventing and controlling the spread of diseases. Therefore, nosemosis detection methods must uphold the highest standards of accuracy to prevent false-negative and false-positive results. We have previously created mAbs for diagnosing *N. ceranae* infections [12] by employing the antibody production technique developed by Kohler and Milstein [18]. Approximately 985 hybridomas were screened, of which three monoclones, namely 9A4, 14A8, and 19B2, demonstrated a robust reaction with the spore antigen of *N. ceranae*. This method significantly enhanced targeting of *Nosema* spores. These three mAbs facilitated the identification of *N. ceranae* spore lysate with a concentration level > 1 μg using Western blotting. 

Herein, we assessed the efficacy of 9A4, 14A8, and 19B2 in targeting *N. ceranae* spores using a sandwich ELISA approach. An ELISA technique utilizing mAbs for detecting *Nosema bombycis* (*N. bombycis*) spores has been previously developed [19,20]; however, this method has shown significant limitations in its sensitivity for detecting antigens. Kawarabata and Hayasaka [21] have also developed a highly sensitive ELISA method capable of detecting as little as 600 ng/mL of soluble protein from the lysate of *N. bombycis* spores. Moreover, Aronstein et al. [22] created polyclonal antibodies based on a single protein (SWP-32; spore wall protein of *N. bombycis*) and utilized them in an ELISA approach. The advantage of employing the polyclonal antibody targeting SWP-32 in Western blotting lies in its capacity to specifically detect a single *Nosema* antigen and the shorter sample preparation time required than that required for *Nosema* detection using PCR. Addressing the limitations associated with polyclonal antibodies is challenging; therefore, in this study, we developed a sandwich ELISA method using biotinylated mAbs to enhance antigen detection activity. Moreover, self-sandwiching antibody pairs (19B2 and biotinylated-19B2, and 14A8 and biotinylated-14A8) showed potent sensitivities. Self-sandwiching antibody pairs can be employed when the target antigen possesses sufficient size to present multiple binding sites or epitopes at appropriate intervals, facilitating effective capture and detection. The self-sandwich method is better suited for cases where the amount of antigen is small [23]. Our findings revealed that the sandwich ELISA method allowed for detection at a minimum concentration of 156 ng/mL, surpassing previously reported detection limits [9]. 

ICG is commonly employed for early identification and initial screening of infectious diseases before conducting confirmatory real-time PCR tests [24,25]. Antigen tests provide rapid results and are user-friendly, allowing for extensive and swift screening of potential infections on a large scale. Real-time PCR is the most commonly used method for detecting *Nosema* infections. Although this method offers precise data regarding the infection status, the sample preparation process is time-consuming and complicated. In our previous study, mAbs were developed for Western blotting assays. The sample preparation procedures for these experimental techniques are simpler and faster than those for real-time PCR; however, they require longer overall experimental time and show lower accuracy. The ICG method introduced in this study for *Nosema* spore detection offers a relatively shorter total experimental time, including sample preparation, than that of Western blotting and ELISA methods. The minimum detection concentration of the ICG was 440 ng/mL, which was higher than that using the sandwich ELISA method (156 ng/mL) in this study. While the diagnostic activity of ICG for *Nosema* infection may be lower than that of ELISA, it offers the distinct advantage of being faster and more convenient. To date, no attempts have been made to develop an ICG for diagnosing *Nosema*. 

This study has some limitations. The ICG utilized in this study was prepared using antibodies initially developed for Western blot analysis; therefore, whether they are suitable for achieving optimal ICG results is not definitive. While Western blotting and ICG rely on antigen-antibody interactions for analysis, the procedures for antigen detection and signal generator (i.e., gold colloid) are distinct. In addition to the binding activity of antibodies against antigen, factors that may influence the potential of false results in ICG include sample diluent composition, protein extraction method used for sample preparation, inadequate specimen collection, logistical and storage conditions of the test, and potential laboratory errors [26,27]. Therefore, to design an ICG with optimal performance, selecting the most suitable antibody combination from a wide array of antibodies is imperative. Further studies are warranted to determine the optimal sample diluent composition and experimental methods.

## 5. Conclusions

The results from the sandwich ELISA demonstrated that combining “19B2 and biotinylated-19B2” exhibited the highest binding activity against antigens. Furthermore, the detection limit of the sandwich ELISA surpassed those previously reported using Western blotting. The establishment of a sandwich ELISA method for the diagnosis of *Nosema* infection is expected to be more precise and reliable than conventional diagnostic methods that rely on microscopic observation. Herein, the sandwich ELISA was superior to ICG, which was designed using the same antibody combination as that used for the sandwich ELISA in diagnosing *Nosema* infection, necessitating further research to optimize the performance of the ICG. The pioneering diagnostic strategies presented in this study will help facilitate the efficient, reliable, and rapid detection of nosemosis, promoting the timely identification and comprehensive management of nosemosis outbreaks.

## Figures and Tables

**Table 1:** Effect of biotinylation on mAb binding activity for *Nosema* spore lysate.

Coated Antigen		*Nosema* Spore Lysate (1 μg/mL)					
Primary Antibody		9A4	14A8	19B2	9A4-biotin	14A8-biotin	19B2-biotin
Antibody Concentration (ng/mL)	1000	0.17 ± 0.015 ^1^	1.99 ± 0.028	0.25 ± 0.01	0.27 ± 0.027	1.75 ± 0.296	1.07 ± 0.048
	100	0.11 ± 0.005	0.33 ± 0.023	0.11 ± 0.026	0.24 ± 0.026	0.36 ± 0.047	0.34 ± 0.016
	10	0.11 ± 0.002	0.12 ± 0.002	0.09 ± 0.015	0.23 ± 0.015	0.26 ± 0.035	0.23 ± 0.012
	0	0.11 ± 0.006	0.09 ± 0.008	0.09 ± 0.012	0.22 ± 0.012	0.23 ± 0.015	0.25 ± 0.036
2nd Antibody		Anti-mouse IgG Fc-HRP (1:10,000)			Avidin-HRP (1:5000)		

^1^ All experiments were repeated more than thrice. Values are presented as means ± standard deviation (n = 3).

**Table 2:** Effect of biotinylation on mAb binding activity for *Nosema* whole spore.

Coated Antigen		*Nosema* Spore (1 × 10^5^ Cells/mL)					
Primary Antibody		9A4	14A8	19B2	9A4-biotin	14A8-biotin	19B2-biotin
Antibody Concentration (ng/mL)	1000	0.12 ± 0.011 ^1^	0.37 ± 0.037	0.14 ± 0.007	0.22 ± 0.024	0.51 ± 0.021	0.42 ± 0.036
	100	0.1 ± 0.012	0.11 ± 0.007	0.08 ± 0.003	0.19 ± 0.017	0.25 ± 0.033	0.25 ± 0.015
	10	0.08 ± 0.001	0.08 ± 0.004	0.07 ± 0.006	0.18 ± 0.011	0.22 ± 0.013	0.26 ± 0.03
	0	0.08 ± 0.003	0.09 ± 0.008	0.08 ± 0.004	0.21 ± 0.013	0.2 ± 0.003	0.19 ± 0.002
2nd Antibody		Anti-mouse IgG Fc-HRP (1:10,000)			Avidin-HRP (1:5000)		

^1^ All experiments were repeated more than thrice. Values are presented as means ± standard deviation (n = 3).

**Table 3:** Detection of *Nosema* antigen (250 and 1000 ng/mL) using a combination of biotinylated and unbiotinylated mAbs (1 μg/mL) in sandwich ELISA.

**Capture Antibody**		**9A4 Antibody (1 μg/mL)**		
**Detector Antibody**		**9A4-biotin (1 μg/mL)**	**14A8-biotin (1 μg/mL)**	**19B2-biotin (1 μg/mL)**
*Nosema* spore lysate (ng/mL)	1000	0.20 ± 0.028 ^1^	0.18 ± 0.006	0.22 ± 0.027
	250	0.18 ± 0.017	0.16 ± 0.003	0.20 ± 0.022
**Capture Antibody**		**14A8 Antibody (1 μg/mL)**		
**Detector Antibody**		**9A4-biotin (1 μg/mL)**	**14A8-biotin (1 μg/mL)**	**19B2-biotin (1 μg/mL)**
*Nosema* spore lysate (ng/mL)	1000	0.20 ± 0.016	0.22 ± 0.020	0.19 ± 0.006
	250	0.18 ± 0.016	0.19 ± 0.008	0.18 ± 0.008
**Capture Antibody**		**19B2 Antibody (1 μg/mL)**		
**Detector Antibody**		**9A4-biotin (1 μg/mL)**	**14A8-biotin (1 μg/mL)**	**19B2-biotin (1 μg/mL)**
*Nosema* spore lysate (ng/mL)	1000	0.18 ± 0.005	0.31 ± 0.096	0.21 ± 0.004
	250	0.15 ± 0.011	0.15 ± 0.006	0.18 ± 0.014

^1^ All experiments were repeated more than thrice. Values are presented as means ± standard deviation (n = 3).

**Table 4:** Detection of *Nosema* antigen (up to 10,000 ng/mL) using a combination of biotinylated and unbiotinylated mAbs (5 μg/mL) in sandwich ELISA.

**Capture Antibody**		**9A4 (5 μg/mL)**		
**Detector Antibody**		**9A4-biotin (1 μg/mL)**	**14A8-biotin (1 μg/mL)**	**19B2-biotin (1 μg/mL)**
*Nosema* spore Lysate (ng/mL)	10,000	0.25 ± 0.029 ^1^	0.39 ± 0.037	0.49 ± 0.011
	2500	0.24 ± 0.010	0.31 ± 0.020	0.31 ± 0.015
	625	0.24 ± 0.018	0.29 ± 0.016	0.26 ± 0.011
	156.3	0.22 ± 0.012	0.29 ± 0.032	0.24 ± 0.013
	0	0.19 ± 0.005	0.26 ± 0.006	0.26 ± 0.015
**Capture Antibody**		**14A8 (5 μg/mL)**		
**Detector Antibody**		**9A4-biotin (1 μg/mL)**	**14A8-biotin (1 μg/mL)**	**19B2-biotin (1 μg/mL)**
*Nosema* spore Lysate (ng/mL)	10,000	0.57 ± 0.020	2.88 ± 0.082	2.32 ± 0.093
	2500	0.37 ± 0.037	1.03 ± 0.021	0.89 ± 0.112
	625	0.32 ± 0.010	0.56 ± 0.050	0.42 ± 0.011
	156.3	0.39 ± 0.033	0.42 ± 0.047	0.34 ± 0.029
	0	0.31 ± 0.017	0.34 ± 0.013	0.29 ± 0.015
**Capture Antibody**		**19B2 (5 μg/mL)**		
**Detector Antibody**		**9A4-biotin (1 μg/mL)**	**14A8-biotin (1 μg/mL)**	**19B2-biotin (1 μg/mL)**
*Nosema* spore Lysate (ng/mL)	10,000	1.69 ± 0.090	3.04 ± 0.051	3.05 ± 0.055
	2500	0.59 ± 0.020	2.53 ± 0.348	2.65 ± 0.117
	625	0.33 ± 0.011	0.97 ± 0.063	1.43 ± 0.088
	156.3	0.26 ± 0.015	0.39 ± 0.011	0.50 ± 0.038
	0	0.14 ± 0.052	0.23 ± 0.012	0.21 ± 0.007

^1^ All experiments were repeated more than thrice. Values are presented as means ± standard deviation (n = 3).

## Data Availability

The data presented in this study are available on request from the corresponding author. The data are not publicly available due to the excessive data size.

## References

[1] Fries I., Feng F., Dasilva A., Slemenda S.B., Pieniazek N.J. (1996). Nosema ceranae n sp (Microspora, Nosematidae), morphological and molecular characterization of a microsporidian parasite of the Asian honey bee Apis cerana (Hymenoptera, Apidae). Eur. J. Protistol..

[2] Higes M., Martin-Hernandez R., Botias C., Bailon E.G., Gonzalez-Porto A.V., Barrios L., Del Nozal M.J., Bernal J.L., Jimenez J.J., Palencia P.G. (2008). How natural infection by Nosema ceranae causes honeybee colony collapse. Environ. Microbiol..

[3] Klee J., Besana A.M., Genersch E., Gisder S., Nanetti A., Tam D.Q., Chinh T.X., Puerta F., Ruz J.M., Kryger P. (2007). Widespread dispersal of the microsporidian Nosema ceranae, an emergent pathogen of the western honey bee, Apis mellifera. J. Invertebr. Pathol..

[4] Paxton R.J., Klee J., Korpela S., Fries I. (2007). *Nosema ceranae* has infected *Apis mellifera* in Europe since at least 1998 and may be more virulent than *Nosema apis*. Apidologie.

[5] Fries I. (2010). *Nosema ceranae* in European honey bees (*Apis mellifera*). J. Invertebr. Pathol..

[6] Martín-Hernández R., Botías C., Bailón E.G., Martínez-Salvador A., Prieto L., Meana A., Higes M. (2012). Microsporidia infecting *Apis mellifera*: Coexistence or competition. Is *Nosema ceranae* replacing *Nosema apis*?. Environ. Microbiol..

[7] Michalczyk M., Sokot R. (2014). Nosemosis in honey bees. Pol. J. Nat. Sci..

[8] Cantwell G.E. (1970). Standard methods for counting nosema spores. Am. Bee J..

[9] Kim J.H., Park J.K., Lee J.K. (2016). Evaluation of antimicrosporidian activity of plant extracts on *Nosema ceranae*. J. Apic. Sci..

[10] Klee J., Tek Tay W., Paxton R.J. (2006). Specific and sensitive detection of *Nosema bombi* (Microsporidia: Nosematidae) in bumble bees (Bombus spp.; Hymenoptera: Apidae) by PCR of partial rRNA gene sequences. J. Invertebr. Pathol..

[11] Carletto J., Blanchard P., Gauthier A., Schurr F., Chauzat M.P., Ribiere M. (2013). Improving molecular discrimination of Nosema apis and Nosema ceranae. J. Invertebr. Pathol..

[12] Kim D.Y., Lee J.K. (2021). Development of monoclonal antibodies against spores of Nosema ceranae for the diagnosis of nosemosis. J. Apic. Res..

[13] Gisder S., Mockel N., Linde A., Genersch E. (2011). A cell culture model for *Nosema ceranae* and *Nosema apis* allows new insights into the life cycle of these important honey bee-pathogenic microsporidia. Environ. Microbiol..

[14] Gisder S., Hedtke K., Mockel N., Frielitz M.C., Linde A., Genersch E. (2010). Five-year cohort study of *Nosema* spp. in Germany: Does climate shape virulence and assertiveness of *Nosema ceranae*?. Appl. Environ. Microbiol..

[15] Genersch E., Evans J.D., Fries I. (2010). Honey bee disease overview. J. Invertebr. Pathol..

[16] Anuracpreeda P., Wanichanon C., Chawengkirtikul R., Chaithirayanon K., Sobhon P. (2009). Fasciola gigantica: Immunodiagnosis of fasciolosis by detection of circulating 28.5 kDa tegumental antigen. Exp. Parasitol..

[17] Laemmli U.K. (1970). Cleavage of structural proteins during the assembly of the head of bacteriophage T4. Nautre.

[18] Kohler G., Milstein C. (1975). Continuous cultures of fused cells secreting antibody of predefined specificity. Nature.

[19] Qian Y., Guo X., Hu X. (1986). Distinguishing the Pebrine spores *N. bombycis*, of the mulberry silkworm by immunoenzymatic technique. Sci. Seric..

[20] Greenstone M.H. (1983). An Enzyme-Linked Immunosorbent-Assay for the Amblyospora Sp of Culex-Salinarius (Microspora, Amblyosporidae). J. Invertebr. Pathol..

[21] Kawarabata T., Hayasaka S. (1978). An Enzyme-Linked-Immunosorbent-Assay to Detect Alkali-Soluble Spore Surface-Antigens of Strains of Nosema-Bombycis (Microspora, Nosematidae). J. Invertebr. Pathol..

[22] Aronstein K.A., Webster T.C., Saldivar E. (2013). A serological method for detection of Nosema ceranae. J. Appl. Microbiol..

[23] Hsu S.-M., Ree H.J. (1980). Self-sandwich Method. An Improved Immunoperoxidase Technic for the Detection of Small Amounts of Antigens. Am. J. Clin. Pathol..

[24] Mertens P., De Vos N., Martiny D., Jassoy C., Mirazimi A., Cuypers L., Van den Wijngaert S., Monteil V., Melin P., Stoffels K. (2020). Development and potential usefulness of the COVID-19 Ag Respi-Strip diagnostic assay in a pandemic context. Front. Med..

[25] Korenkov M., Poopalasingam N., Madler M., Vanshylla K., Eggeling R., Wirtz M., Fish I., Dewald F., Gieselmann L., Lehmann C. (2021). Evaluation of a rapid antigen test to detect SARS-CoV-2 infection and identify potentially infectious individuals. J. Clin. Microbiol..

[26] Lippi G., Simundic A.-M., Plebani M. (2020). Potential preanalytical and analytical vulnerabilities in the laboratory diagnosis of coronavirus disease 2019 (COVID-19). Clin. Chem. Lab. Med..

[27] Richard-Greenblatt M., Ziegler M.J., Bromberg V., Huang E., Abdallah H., Tolomeo P., Lautenbach E., Glaser L., Kelly B.J. (2020). Impact of nasopharyngeal specimen quality on SARS-CoV-2 test sensitivity. Med. Rxiv..

